# Errata

**DOI:** 10.36660/abc.20250868

**Published:** 2026-03-17

Edição de Fevereiro de 2024, vol. 121(2):e20240125

No Minieditortial “Relação entre Complacência Pulmonar Estática e Extubação após Cirurgia Cardíaca”, com número de DOI: https://doi.org/10.36660/abc.20240125, publicado no periódico Arquivos Brasileiros de Cardiologia, Arq Bras Cardiol. 2024; 121(2):e20240125, na página 1, alterar o parágrafo “Parte do material deste artigo integrou a dissertação de mestrado da Dra. Thais da Silva Brito, realizada sob a orientação da Professora Doutora Desanka Dragosavac, na Universidade Estadual de Campinas.^2^” para “Parte do material deste artigo integrou a dissertação de mestrado da Dra. Thais Bento Rudge Ramos, realizada sob a orientação da Professora Doutora Desanka Dragosavac, na Universidade Estadual de Campinas.^2^”.

Edição de Julho de 2025, vol. 122(7):e20240845

No Artigo Original “Ablação por Campo Pulsado versus Ablação por Radiofrequência de Curta Duração e Potência Muito Alta na Fibrilação Atrial: Uma Revisão Sistemática e Metanálise”, com número de DOI: https://doi.org/10.36660/abc.20240845, publicado no periódico Arquivos Brasileiros de Cardiologia, Arq Bras Cardiol. 2025; 122(7):e20240845, na página 1, alterar o nome do autor “Juan Alejandro Pinilla Alarcón“ para “Juan Pinilla”.

Edição de Outubro de 2025, vol. 122(10):e20250151

No Artigo Original “Utilidade Diagnóstica da Duração da Onda P na Identificação de Sobrecarga Atrial Esquerda: Lições da Coorte Elsa-Brasil”, com número de DOI: https://doi.org/10.36660/abc.20250151, publicado no periódico Arquivos Brasileiros de Cardiologia, Arq Bras Cardiol. 2025; 122(10):e20250151, na página 8, alterar a referência 13 “Alencar JN Neto. Tratado de ECG. Salvador: Sanar; 2022” para “Pastore CA, Samesima N, Tobias N, Pereira Filho HG, organizadores. Eletrocardiografia atual: curso de eletrocardiografia do InCor. São Paulo: Editora Atheneu; 2016.”

Edição de Novembro de 2025, vol. 122(11):e20250176

No Artigo Original “Associação da Osmolalidade Plasmática com o Risco de Doença Cardiovascular: Evidências do Banco de Dados do National Health and Nutrition Examination Survey”, com número de DOI: https://doi.org/10.36660/abc.20250176, publicado no periódico Arquivos Brasileiros de Cardiologia, Arq Bras Cardiol. 2025; 122(11):e20250176, na página 1, alterar a instituição “Nanjing University of Traditional Chinese Medicine Affiliated Wuxi Hospital - Cardiovascular” para “Wuxi Affiliated Hospital of Nanjing University of Chinese Medicine - Cardiovascular”.

