# Supplementary material for: Errata
Source: Arq Bras Cardiol. 2026 Mar 17;123(2):e20250868. [Article in Portuguese] doi: 10.36660/abc.20250868 (PMC13148857; doi:10.36660/abc.20250868)
Supplement: Translation

# Translation: Erratum

**DOI:** 10.36660/abc.20250868i

February 2024 Issue, vol. 121(2):e20240125

In the Short Editorial “Relationship between Static Lung Compliance and Extubation After Cardiac Surgery”, with DOI: https://doi.org/10.36660/abc.20240125, published in the journal Arquivos Brasileiros de Cardiologia, Arq Bras Cardiol. 2024; 121(2):e20240125, on page 1, change the paragraph “This paper is part of the MSc thesis of Dr. Thais da Silva Bento, under the orientation of Dr. Desanka Dragosavac at the Universidade Estadual de Campinas^2^.” to “This paper is part of the MSc thesis of Dr. Thais Bento Rudge Ramos, under the orientation of Dr. Desanka Dragosavac at the Universidade Estadual de Campinas.^2^”.

July 2025 Issue, vol. 122(7):e20240845

In the Original Article “Pulsed Field Ablation Versus Very High-Power Short-Duration Radiofrequency Ablation in Atrial Fibrillation: A Systematic Review and Meta-Analysis”, with DOI: https://doi.org/10.36660/abc.20240845i, published in the journal Arquivos Brasileiros de Cardiologia, Arq Bras Cardiol. 2025; 122(7):e20240845, on page 1, change the name of the author “Juan Alejandro Pinilla Alarcón” to “Juan Pinilla”.

October 2025 Issue, vol. 122(10):e20250151

In the Original Article “Diagnostic Utility of P-Wave Duration in Identifying Left Atrial Enlargement: Insights from the ELSA-Brasil Cohort”, with DOI: https://doi.org/10.36660/abc.20250151, published in the journal Arquivos Brasileiros de Cardiologia, Arq Bras Cardiol. 2025; 122(10):e20250151, on page 7, change reference 13 “Alencar JN Neto. Tratado de ECG. Salvador: Sanar; 2022” to “Pastore CA, Samesima N, Tobias N, Pereira Filho HG, organizadores. Eletrocardiografia atual: curso de eletrocardiografia do InCor. São Paulo: Editora Atheneu; 2016.”

November 2025 Issue, vol. 122(11):e20250176

In the Original Article “Association of Plasma Osmolality with Cardiovascular Disease Risk: Evidence from the National Health and Nutrition Examination Survey Database”, with DOI: https://doi.org/10.36660/abc.20250176, published in the journal Arquivos Brasileiros de Cardiologia, Arq Bras Cardiol. 2025; 122(11):e20250176, on page 1, change the institution “Nanjing University of Traditional Chinese Medicine Affiliated Wuxi Hospital - Cardiovascular” to “Wuxi Affiliated Hospital of Nanjing University of Chinese Medicine - Cardiovascular”.
